# Cross‐cultural adaptation, validity and reliability of the Arabic version of the Penn Shoulder Score

**DOI:** 10.1002/jeo2.70982

**Published:** 2026-11-13

**Authors:** Waleed Albishi, Omar A. Aldosari, Mohammed N. Alhuqbani, Zyad A. Aldosari, Hisham Alsanawi, Abdulrahman Alaseem, Emad Elansary

**Affiliations:** ^1^ Department of Orthopedic Surgery, College of Medicine King Saud University Riyadh Saudi Arabia; ^2^ Ministry of Health East Jeddah Hospital Jeddah Saudi Arabia

**Keywords:** Arabic, outcome measures, Penn Shoulder Score, PSS, shoulder

## Abstract

**Purpose:**

This study aimed to translate, culturally adapt and validate the Arabic version of the Penn Shoulder Score (Ar‐PSS) for assessing shoulder dysfunction among Arabic‐speaking populations.

**Methods:**

The translation and cultural adaptation process strictly followed internationally recognized guidelines, ensuring linguistic and cultural appropriateness. The reliability of the Ar‐PSS was assessed through internal consistency (Cronbach's *α*) and test–retest reliability (intraclass correlation coefficient, ICC). The standard error of measurement (SEM) and minimal detectable change (MDC) were calculated to determine measurement precision. Construct validity was evaluated through correlations with the Disabilities of the Arm, Shoulder and Hand Scale (DASH) and the 36‐Item Short Form Health Survey (SF‐36) health survey. Additionally, Bland–Altman analysis was performed to assess measurement agreement.

**Results:**

A total of 80 participants with shoulder disorders completed the baseline assessment. Forty participants completed the retest assessment after a 3‐week interval. The Ar‐PSS demonstrated excellent internal consistency (Cronbach's *α* = 0.94) and test–retest reliability (ICC = 0.88). The SEM was 2.92, and the MDC was 8.09, indicating acceptable measurement precision and sensitivity. Significant correlations were observed with DASH (*r* = −0.67, *p* < 0.01) and SF‐36 Pain (*r* = 0.53, *p* < 0.01). Bland–Altman analysis revealed no proportional bias, affirming the accuracy and consistency of the Ar‐PSS.

**Conclusion:**

The Ar‐PSS has demonstrated robust reliability, validity and cultural suitability for evaluating shoulder dysfunction among Arabic‐speaking populations and provides an additional validated shoulder‐specific outcome measure for Arabic‐speaking populations.

**Level of Evidence:**

Level II, diagnostic study (instrument validation).

**Abbreviations**
Ar‐PSS: Arabic Penn Shoulder Score
ASES: American Shoulder and Elbow Surgeons Evaluation Form
DASH: Disabilities of the Arm, Shoulder and Hand
ICC: intraclass correlation coefficient
IRB: institutional review board
MDC: minimal detectable change
OSS: Oxford Shoulder Score
PSS: Penn Shoulder Score
ROM: range of motion
SD: standard deviation
SEM: standard error of measurement
SF‐36: 36‐Item Short Form Health Survey
SPADI: Shoulder Pain and Disability Index
SPSS: Statistical Package for the Social Sciences
UCLA: University of California Los Angeles Shoulder Rating Scale
WOOS: Western Ontario Osteoarthritis of the Shoulder Index
WORC: Western Ontario Rotator Cuff Index
WOSI: Western Ontario Shoulder Instability Index

## INTRODUCTION

Shoulder dysfunction is a common cause of musculoskeletal pain affecting about 20%–33% of the general population [19]. Pain and reduced range of motion (ROM) due to various shoulder disorders are significantly associated with increased physical infirmity, impacting work activities and the quality of life [5, 8]. Intervention in those patients aims to restore shoulder function while controlling pain to achieve painless ROM [20]. Therefore, traditional objective and even subjective measurement tools are equally important [25] to quantify a primary outcome in assessing shoulder function, deformity progression or treatment efficacy [21].

Physicians usually depend on their physical examination to assess ROM and muscle strength; nonetheless, further quantifying the degree of disability and pain is needed using validated subjective scales [21, 25]. There are many tools available in English, but no gold standard is universally accepted [10]. Disabilities of the Arm, Shoulder and Hand Scale (DASH) [12], American Shoulder and Elbow Surgeons Evaluation Form (ASES) [23], the Constant Score [7], the Shoulder Pain and Disability Index (SPADI) [24], the Oxford Shoulder Score (OSS) [9], the Western Ontario Shoulder Tools (the Western Ontario Shoulder Instability Index [WOSI], the Western Ontario Osteoarthritis of the Shoulder Index [WOOS] and the Western Ontario Rotator Cuff Index [WORC]) [14, 15, 18], the Simple Shoulder Test [17], University of California Los Angeles Shoulder Rating Scale [4] and the Penn Shoulder Score (PSS) [19] are valid, reliable and widely used scales. However, although many tools are available to assess shoulder disability, they cannot be generalized without validation and cultural adaptation to account for language and social culture [6].

PSS is a specific self‐report measure developed by Leggin et al. [16] in 1999 to evaluate shoulder dysfunctions, using a 100‐point scale that assesses three shoulder subscale domains: pain, patient satisfaction and function. The total PSS score ranges from 0 to 100. One hundred points indicate a high function, low pain and high satisfaction with shoulder function [16]. This tool needs to be translated, validated and culturally adapted to increase its use. To our knowledge, the only tools validated in Arabic to be used for shoulder dysfunction are Arabic DASH [1], Arabic SPADI [2] and Arabic OSS [3]. Several shoulder PROMs have been translated into Arabic; however, the PSS uniquely combines pain, satisfaction and function within a single instrument. The translation needs to follow a cross‐cultural adaptation process to decrease the risk of bias in a study and give a broader perspective of shoulder disorders [11, 22]. This study aimed to evaluate the internal consistency, test–retest reliability and construct validity of the translated and adapted Arabic version of the PSS scales.

## METHODS

### Study design, participants and setting

Our study was conducted in the orthopaedic outpatient clinics at King Saud University Medical City. Our inclusion criteria were all adults aged 18 and above who spoke, read and wrote Arabic and had shoulder pathology, including arthritis and fractures, among patients seen in our orthopaedic clinic, as well as other shoulder injuries. Regarding our exclusion criteria, we excluded patients with a history of neurological disease, neck pathology, rheumatoid arthritis, diabetes mellitus, inflammatory joint disease or those unable to understand Arabic. The study cohort consisted of 80 participants with shoulder disorders. Rotator cuff pathology was the predominant diagnosis, accounting for 55 participants (68.8%), while 25 participants (31.2%) were diagnosed with shoulder instability. Diagnoses were established by fellowship‐trained orthopaedic surgeons based on clinical evaluation and imaging findings when indicated.

### Instrument translation, procedure and data collection

Our study consisted of two stages: The first stage involved translating the PSS from English into Arabic. The second stage involved data collection from the selected sample to assess cross‐cultural adaptivity and reliability. Written reports were compiled at each stage of the cultural adaptation process, detailing all versions of the questionnaire along with the corresponding modifications. During the validation phase, an electronic survey format was employed. The translation process adhered to the guidelines set by the MAPI Research Trust [16], encompassing four key stages: forward translation, back translation, pilot testing and final proofreading.

#### Translation process

The translation and cultural adaptation of the PSS followed internationally accepted cross‐cultural adaptation guidelines as described by Sousa and Rojjanasrirat [25] and is illustrated in Figure 1.

**Figure 1:**
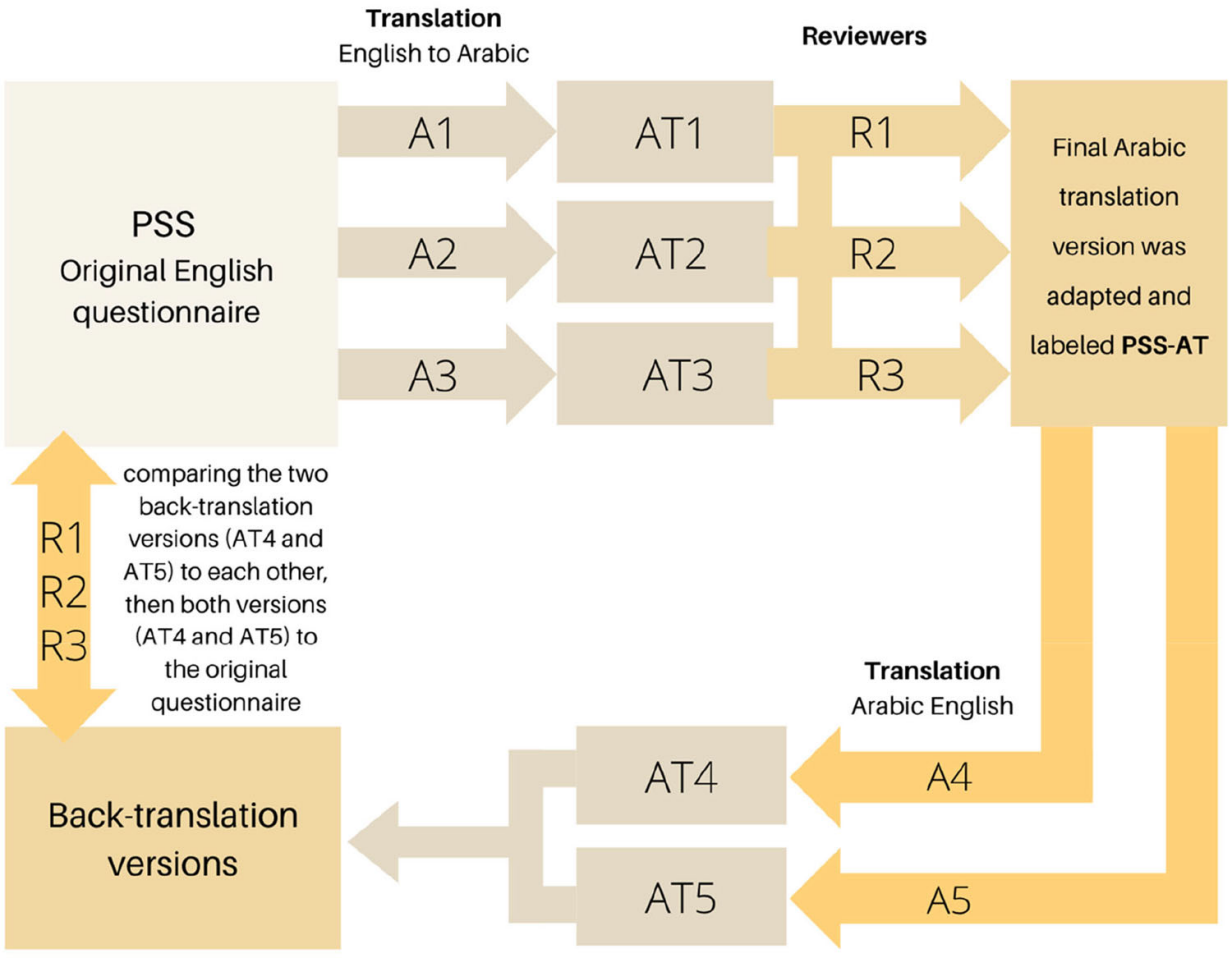
Translation and the cultural adaptation of the PSS scale stages. PSS, Penn Shoulder Score.

##### Forward translation

The original English version of the PSS was independently translated into Arabic by three bilingual translators (AT1, AT2 and AT3) whose native language was Arabic. Two translators had medical backgrounds, while the third had no healthcare background, ensuring both conceptual and lay‐language equivalence.

##### Reconciliation

The three forward‐translated versions were reviewed and compared by an expert committee consisting of three independent reviewers (R1, R2 and R3). Discrepancies were discussed until consensus was reached, resulting in a single reconciled Arabic version (PSS‐AT).

##### Back translation

The reconciled Arabic version was independently back‐translated into English by two bilingual translators (AT4 and AT5) whose native language was English and who were blinded to the original PSS questionnaire.

##### Expert committee review

The expert committee (R1, R2 and R3) compared the two back‐translated versions with the original English questionnaire to evaluate semantic, idiomatic, experiential and conceptual equivalence. No major discrepancies were identified, and minor linguistic refinements were made where necessary.

##### Pilot testing

The pre‐final Arabic version was pilot‐tested in 10 native Arabic‐speaking participants with shoulder pathology. Participants were interviewed regarding the clarity, comprehensibility and cultural relevance of each item. Any comments or suggestions were reviewed by the expert committee.

##### Finalization

Following pilot testing and expert committee review, the final Arabic version of the Penn Shoulder Score (Ar‐PSS) was produced and approved for psychometric validation.

#### Validation process and data acquisition

A pilot study was conducted with 10 participants to evaluate the clarity and comprehensibility of the Arabic‐translated PSS questionnaire prior to initiating full‐scale data collection. The panel included native Arabic‐speaking individuals (six men and four women) aged between 20 and 45 years, all of whom presented with various shoulder pathologies. During one‐on‐one interviews, the interviewer assessed participants' understanding by asking whether they encountered any difficulties interpreting the questions. For any items deemed unclear, alternative translations were suggested. The PSS‐AT version incorporated final modifications and was subsequently reviewed by all the reviewers. The finalized version was titled the Arabic Penn Shoulder Score (Ar‐PSS).

A cross‐sectional study was conducted over 4 months. From a pool of eligible patients attending an outpatient orthopaedic clinic, 80 native Arabic‐speaking individuals were recruited consecutively, following the same approach as the pilot study. Participants were asked to complete the Arabic version of the PSS (Ar‐PSS) questionnaire in two sessions spaced 3 weeks apart to evaluate the instrument's reliability. In the first session, participants completed the Ar‐PSS questionnaire. In the second session, conducted 3 weeks later, they completed the Ar‐PSS along with the DASH and 36‐Item Short Form Health Survey (SF‐36) questionnaires [22, 25] to assess construct validity. The evaluation of reliability and validity was guided by widely accepted psychometric standards [25]. Participants completed the retest assessment over a relatively short 3‐week interval, during which no surgical intervention or major change in clinical management was planned. Participants were also asked whether they had experienced any substantial change in their shoulder symptoms prior to completing the second assessment, and no clinically meaningful changes were reported.

### Statistical data analysis

Data were analysed using the Statistical Package for the Social Sciences (SPSS 22; IBM Corp.). Questionnaire reliability was assessed using Cronbach's *α*, while test–retest reliability of the Ar‐PSS was evaluated through intraclass correlation coefficients (ICCs). Test–retest reliability was evaluated using a two‐way random‐effects model with absolute agreement for single measurements (ICC(2,1)), in accordance with current recommendations for reporting reliability studies. Spearman's correlation coefficient was employed to assess construct validity. Test–retest agreement was further examined using a Bland‐Altman plot. Statistical significance was set at *p* < 0.05. Measurement agreement was evaluated using the standard error of measurement (SEM) and the minimal detectable change (MDC). The SEM, reflecting measurement precision, was calculated as SEM = SD × √(1 − ICC). The MDC, representing the smallest change beyond measurement error, was calculated at the 95% confidence level using the formula: MDC_95_ = 1.96 × √2 × SEM.

## RESULTS

Eighty patients (80) participated in the initial survey. To assess test–retest reliability, forty (40) participants were randomly selected for a follow‐up assessment. This subgroup was demographically and clinically comparable to the overall sample. All 40 participants completed the retest, which included the Ar‐PSS, as well as the DASH and SF‐36 questionnaires. The mean interval between test and retest assessments was 21 days. The mean age of participants during the initial survey was 30.6 ± 7.7 years, with males comprising the majority (88.8%). All participants reported no difficulties in understanding any part of the Ar‐PSS during the first administration, and every item achieved a complete response rate. Table 1 displays the absolute scores for all three instruments completed during the retest.

**Table 1:** Descriptive characteristics of all scores.

Scales	Mean ± SD	Minimum	Maximum
PSS			
Total Shoulder Score	61.93 ± 8.41	39	73
Pain	8.16 ± 7.36	0	29
Function	47.21 ± 11.91	9	60
Satisfaction	6.55 ± 3.10	0	10
*DASH Disability Score*	41.90 ± 17.59	24.17	83.33
SF‐36			
Physical functioning	74.50 ± 33.30	0	100
Pain	82.13 ± 25.14	20	100
Role physical	65.00 ± 41.53	0	100
General health	67.75 ± 20.44	25	100
Vitality	59.25 ± 21.47	10	100
Role emotional	73.33 ± 42.83	0	100
Mental health	64.70 ± 19.93	20	100
Social functioning	88.13 ± 16.73	37.5	100

Abbreviations: DASH, Disabilities of the Arm, Shoulder and Hand; PSS, Penn Shoulder Score; SD, standard deviation; SF‐36, 36‐Item Short Form Health Survey.

### Validity

Spearman's correlation analysis, as presented in Table 2, demonstrated strong construct validity of the Ar‐PSS. The total score showed a significant negative correlation with the DASH score (*r* = –0.670, *p* < 0.01), with similarly strong correlations observed in the pain, function and satisfaction subscales. The Ar‐PSS also showed moderate to strong positive correlations with several SF‐36 domains, including pain, role physical, general health and social functioning. Figure 2 illustrates a statistically significant negative correlation between the Ar‐PSS total score and the Disability of the Arm, Shoulder and Hand (DASH) score, indicating that higher Ar‐PSS scores—reflecting better shoulder function—are associated with lower DASH scores. Figure 3 demonstrates a significant positive correlation between the Ar‐PSS total score and the pain subscale of the SF‐36, indicating that higher Ar‐PSS scores.

**Table 2:** Construct validity and Spearman's correlation coefficients of PSS and DASH shoulder score and 36‐item Short‐form health survey *N* = 40.

Scale	PSS scores			
	Pain	Function	Satisfaction	Total score
DASH	0.682*	−0.735*	−0.645**	−0.670*
SF‐36				
Physical functioning	−0.383**	0.430**	0.171	0.269
Pain	−0.609*	0.632*	0.602*	0.534*
Role physical	−0.493*	0.520*	0.452*	0.433*
General health	−0.441*	0.537*	0.300	0.404*
Vitality	−0.399**	0.436*	0.419*	0.0393**
Role emotional	−0.373**	0.424*	0.283	0.360**
Mental health	−0.298	0.278	0.230	0.202
Social functioning	−0.434*	0.428*	0.469*	0.386**

*Note*: Higher Ar‐PSS scores indicate better shoulder status, whereas higher DASH scores indicate greater disability.

Abbreviations: DASH, Disabilities of the Arm, Shoulder and Hand; PSS, Penn Shoulder Score; SF‐36, 36‐Item Short Form Health Survey.

* Correlation is significant at the 0.01 level (two‐tailed).

** Correlation is significant at the 0.05 level (two‐tailed).

**Figure 2:**
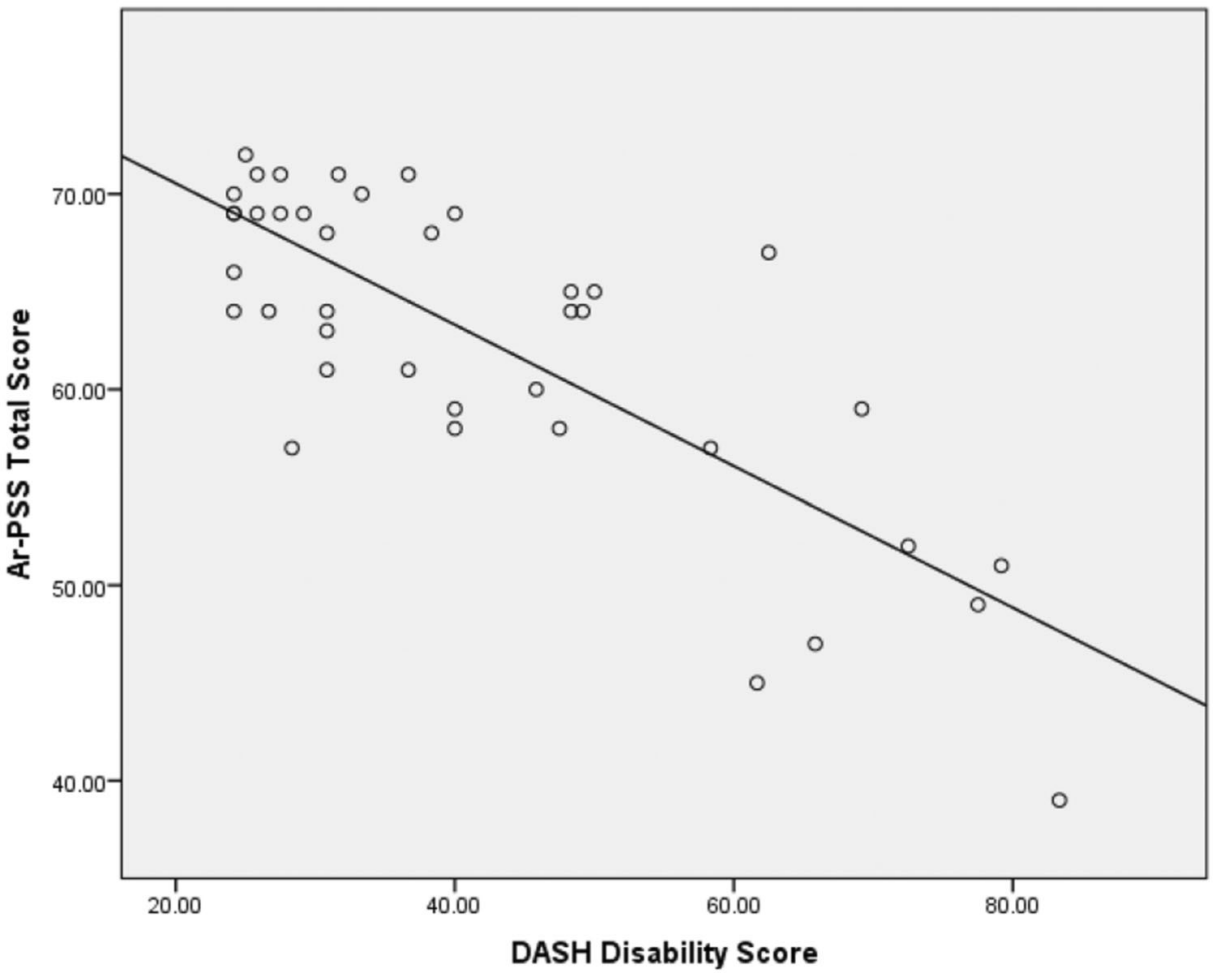
Correlation of the Arabic Penn Shoulder Score (Ar‐PSS) with the Disability of the Arm, Shoulder and Hand (DASH) shoulder score.

**Figure 3:**
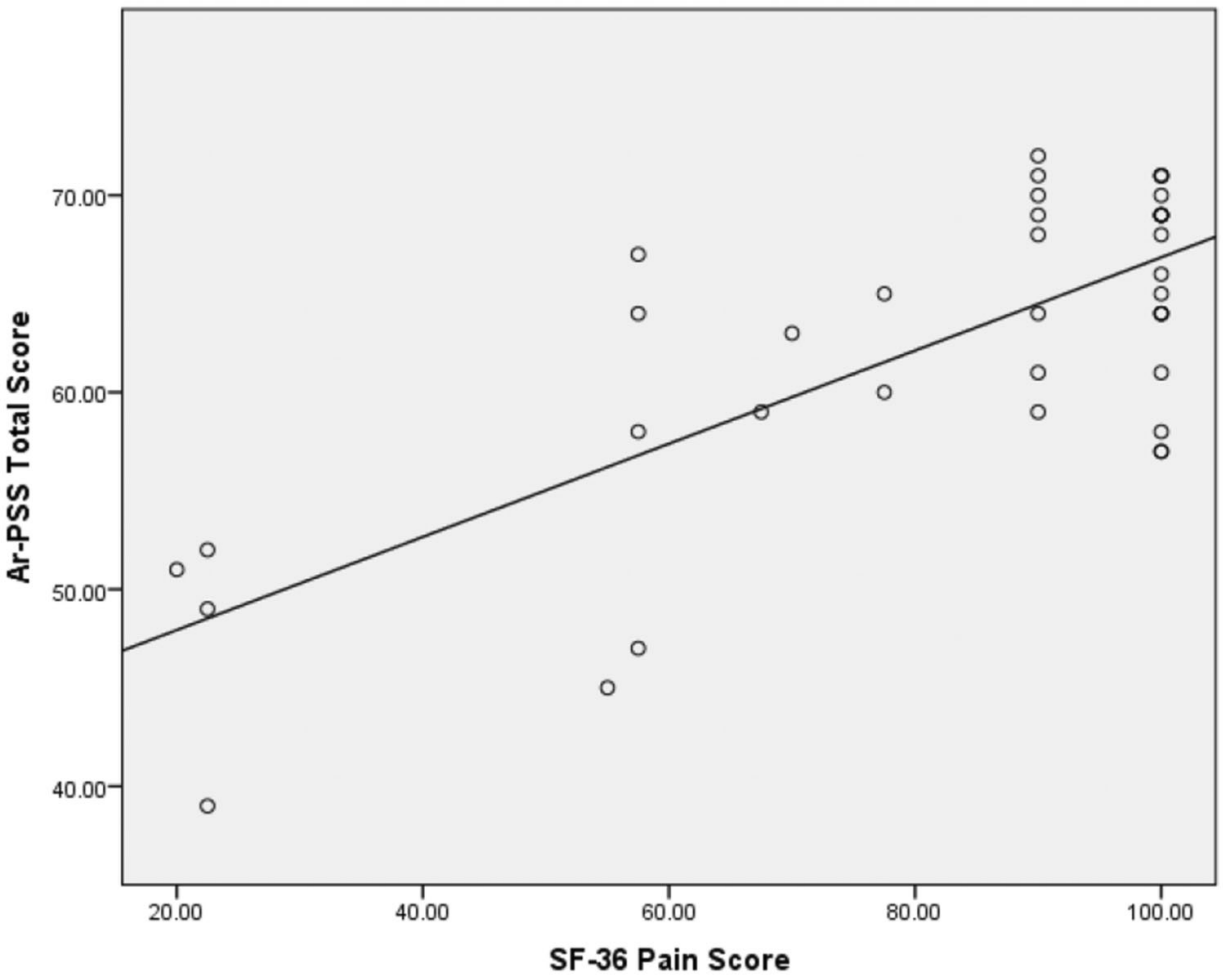
Correlation of the Arabic Penn Shoulder Score (Ar‐PSS) with the pain subscale of the 36‐Item Short Form (SF‐36) health survey.

Table 3 presents the test–retest reliability of the Ar‐PSS items, evaluated using ICCs. ICC values ranged from 0.255 to 0.915, with the majority of items demonstrating good to excellent reliability (ICC ≥ 0.75). Specifically, 18 out of 24 items exceeded an ICC of 0.70, indicating acceptable stability over time. Items 10, 17 and 18 showed the highest reliability (ICC > 0.90), while Item 8 exhibited the lowest reliability (ICC = 0.255).

**Table 3:** Test–retest reliability of the Ar‐PSS, *N* = 40.

Item	Mean ± SD	ICC values	ICC 95% CI	SEM	MDC
1	2.11 ± 2.73	0.420	−0.070 to 0.689	2.08	5.76
2	1.80 ± 2.59	0.818	0.658–0.904	1.10	3.06
3	4.25 ± 3.26	0.810	0.634–0.899	1.42	3.94
4	6.55 ± 3.08	0.835	0.723–0.922	1.25	3.47
5	2.32 ± 0.91	0.805	0.627–0.898	0.40	1.11
6	2.05 ± 0.93	0.897	0.804–0.946	0.30	0.83
7	2.75 ± 0.54	0.789	0.597–0.890	0.25	0.69
8	2.59 ± 0.59	0.255	−0.409 to 0.606	0.51	1.41
9	2.74 ± 0.57	0.771	0.566–0.879	0.27	0.76
10	2.34 ± 0.86	0.915	0.838–0.955	0.25	0.69
11	2.66 ± 0.64	0.722	0.472–0.853	0.34	0.93
12	2.65 ± 0.66	0.724	0.475–0.855	0.35	0.96
13	2.75 ± 0.54	0.798	0.620–0.892	0.24	0.67
14	2.35 ± 0.81	0.733	0.572–0.880	0.42	1.16
15	2.60 ± 0.61	0.647	0.339–0.812	0.36	1.00
16	2.43 ± 0.78	0.879	0.771–0.936	0.27	0.75
17	2.09 ± 0.93	0.908	0.824–0.952	0.28	0.78
18	2.40 ± 0.83	0.898	0.807–0.946	0.27	0.73
19	2.37 ± 0.88	0.848	0.709–0.921	0.34	0.95
20	2.01 ± 0.92	0.876	0.761–0.936	0.32	0.90
21	2.27 ± 0.85	0.666	0.353–0.827	0.49	1.36
22	2.51 ± 0.82	0.821	0.635–0.912	0.35	0.96
23	1.97 ± 0.85	0.881	0.761–0.940	0.29	0.81
24	2.56 ± 0.71	0.656	0.355–0.817	0.42	1.15

*Note*: SEM was calculated as SD × √(1 − ICC), and MDC was calculated as 1.96 × √2 × SEM.

Abbreviations: Ar‐PSS, Arabic Penn Shoulder Score; CI, confidence interval; ICC, intraclass correlation coefficient; MDC, minimal detectable change; SD, standard deviation; SEM, standard error of measurement.

### Reliability

The Ar‐PSS demonstrated excellent internal consistency, with a Cronbach's *α* of 0.94. The overall test–retest reliability was also high, as reflected by an ICC of 0.88, indicating strong reproducibility. The SEM was calculated at 2.92, and the MDC was determined to be 8.09, as presented in Table 3. The Bland–Altman plot shown in Figure 4 was used to assess agreement, and linear regression analysis was performed to evaluate proportional bias. The analysis revealed no statistically significant findings, indicating the absence of proportional bias. The differences between the test and retest were plotted against the mean score. The line indicates the mean difference between the two, ±1.96 standard deviations (SDs).

**Figure 4:**
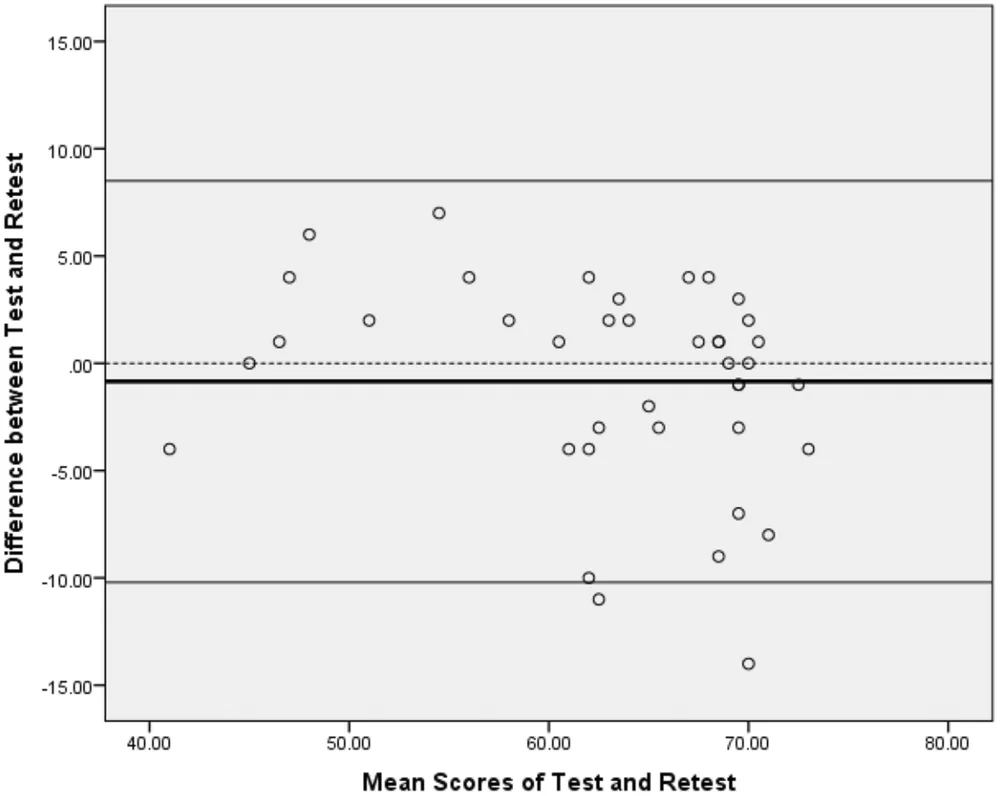
Bland–Altman plot for test–retest agreement of Arabic Penn Shoulder Score (Ar‐PSS) shoulder scale.

## DISCUSSION

The present study translated, culturally adapted and validated the Ar‐PSS. The Ar‐PSS demonstrated excellent internal consistency, strong test–retest reliability and good construct validity against established shoulder outcome measures. Reliability is a fundamental psychometric property for self‐reported measures, reflecting the consistency of the tool. Our study reported a high Cronbach's *α* value of 0.94, demonstrating excellent internal consistency and aligning closely with previously reported values from the original English (*α* = 0.93), Brazilian (*α* = 0.92) and Turkish (*α* = 0.81) versions of the PSS [10, 16, 21]. This suggests that the Ar‐PSS reliably measures the underlying constructs of shoulder pain, satisfaction and function. The test–retest reliability, assessed using the ICC, was also high (ICC = 0.88), indicating outstanding repeatability across testing intervals. This finding parallels ICC values from the original study (ICC = 0.94), the Turkish adaptation (ICC = 0.90) and the Brazilian version (ICC = 0.94), reinforcing the robustness of the scale's reliability [10, 16, 21].

The SEM of the Ar‐PSS was 2.92 points, and the MDC at the 95% confidence level (MDC_95_) was 8.09 points. These metrics provide clinically meaningful information regarding measurement precision and the interpretation of change scores. Specifically, a change greater than 8.09 points on the 100‐point Ar‐PSS scale can be considered a true change beyond measurement error. Compared with the original PSS, which reported an SEM of 8.5 points and an MDC of 12.1 points [16], the SEM and MDC values were comparable to previously reported psychometric properties of the original instrument.

Regarding validity, the construct validity of the Ar‐PSS was assessed through correlations with the DASH and SF‐36 scales. The Ar‐PSS exhibited significant correlations, thus substantiating its construct validity. Such correlations resonate with findings from other language adaptations; for instance, the Turkish PSS demonstrated strong correlation coefficients with Constant (*r* = 0.65), ASES (*r* = 0.78) and WORC (*r* = −0.77), supporting concurrent and convergent validity [10]. Similarly, the Portuguese adaptation underscored the tool's cultural suitability, emphasizing its capability to measure pain, function and satisfaction comprehensively across diverse populations [21]. The Bland–Altman analysis performed in our study further confirmed the absence of proportional bias, affirming the accuracy and consistency of the Ar‐PSS across the range of scores—a critical psychometric feature that aligns with results found in the original and other validated versions [10, 16, 21].

The pain subscale in the Ar‐PSS demonstrated good internal consistency (Cronbach's *α* = 0.812) and test–retest reliability (ICC = 0.809). Correlation analysis revealed a significant inverse correlation with both the DASH pain scale (*r* = −0.682, *p* < 0.01) and SF‐36 pain subscale (*r* = −0.609, *p* < 0.01). Similar to the PSS‐T, the Ar‐PSS utilizes multiple numeric rating scales to evaluate pain across different activity levels, thereby enhancing the sensitivity and reliability of pain assessment compared to single‐item scales employed in the Constant and ASES scores. Numeric rating scales are validated and particularly useful across varied literacy levels, supporting the superiority and versatility of the Ar‐PSS in assessing pain comprehensively [7, 13, 16, 23].

The Ar‐PSS satisfaction subscale showed excellent test–retest reliability (ICC = 0.853). Similar to findings from the PSS‐T, the satisfaction parameter in the Ar‐PSS is unique in that it employs a numeric rating scale, contrasting with binary or absent satisfaction assessments in other popular scales such as ASES, Constant Score and WORC. Unlike these scales, the Ar‐PSS captures satisfaction with a more nuanced quantitative measure, making it potentially more sensitive in assessing patient‐perceived outcomes and overall satisfaction with shoulder function post‐treatment [4, 6, 7, 10, 16, 23].

The function subscale of Ar‐PSS exhibited outstanding reliability (Cronbach's *α* = 0.964; ICC = 0.965) and a strong correlation with the DASH function scale (*r* = −0.735, *p* < 0.01) and SF‐36 physical functioning scale (*r* = 0.430, *p* < 0.05). A similar high correlation was observed in the Turkish version with ASES (*r* = 0.83). Approximately 60% of the Ar‐PSS score comprises functional assessment, surpassing the proportion found in other scales, such as the Constant Score (20%) and is comparable to the ASES (50%). The Ar‐PSS's four‐point Likert scale system effectively excludes activities not performed by the patient, minimizing biases related to cultural or gender differences and thereby enhancing the objectivity and applicability of the functional assessment [7, 16, 23]. Item 8 demonstrated substantially lower test–retest reliability (ICC = 0.255) compared with the remaining items. This may reflect variability in patient interpretation, symptom fluctuation or potential linguistic nuances in the Arabic adaptation.

## LIMITATIONS

The study population was relatively young (mean age 30.6 years) and predominantly male (88.8%). Therefore, caution should be exercised when generalizing these findings to older patients and female populations, particularly those with degenerative shoulder disorders such as rotator cuff disease and glenohumeral osteoarthritis. Responsiveness was not assessed in the current study; therefore, the ability of the Ar‐PSS to detect clinically meaningful changes following treatment remains unknown. Floor and ceiling effects were not formally assessed.

## CONCLUSION

The Ar‐PSS is a reliable, valid and culturally appropriate tool for assessing shoulder dysfunction in Arabic‐speaking populations. It effectively complements existing instruments, addressing the previously identified gap for specific shoulder dysfunction measures in Arabic. Future research should evaluate the responsiveness and broader applicability of the Ar‐PSS across diverse Arabic‐speaking populations.

## AUTHOR CONTRIBUTIONS

Waleed Albishi supervised the study. All authors designed the study. All authors collected and analysed the data. All authors participated in writing the manuscript. All authors read and approved the final manuscript.

## FUNDING

The authors have no funding to report.

## CONFLICT OF INTEREST STATEMENT

The authors declare no conflicts of interest.

## ETHICS STATEMENT

Approval (No. E‐22‐6969) was obtained from the Institutional Review Board (IRB) in the College of Medicine, King Saud University, Riyadh, Saudi Arabia. Each participant provided verbal approval after being informed of the study's purpose and their right to withdraw at any time without any obligation to the study team. Additionally, participants' anonymity was ensured by not collecting identifying data; all participants remained anonymous. No incentives or rewards were given to the participants.

## Data Availability

The datasets utilized in this study can be obtained from the corresponding author upon a reasonable request.
